# Modeling effects of GABAA receptors in basal ganglia computational models

**DOI:** 10.1186/1471-2202-12-S1-P106

**Published:** 2011-07-18

**Authors:** Félix Njap, Andréas Moser, Ulrich Hofmann

**Affiliations:** 1 Institute for Signal Processing, University of Lübeck, Lübeck, D-23538, Germany; 2 Graduate School for Computing medicine and Life Sciences, University of Lübeck, D-23538, Germany; 3 Department of Neurology, University of Lübeck, Lübeck, D-23538, Germany

## 

γ-Aminobutyric acid (GABA) is a major inhibitory neurotransmitter in neurons of the basal ganglia. Recent experimental studies demonstrate that high frequency stimulation (HFS) does not only need presynaptic GABAA receptors but also intact GABAergic nerve terminals coupled to GABAA receptors to exert an inhibitory effect [1-3]. This effect may result to the modification of basal ganglia activity, exactly how has still not been clearly determined. Using a computational approach, our current contribution analyze the firing patterns of different synaptic conductances input applied to the subthalamic nucleus (STN) neuron in Parkinson’s disease (PD) state and compare this to the normal state. Our contribution is based on Rubin and Terman’s PD computational proposed model [4]. To carefully examine the similarity or dissimilarity between both firing patterns, we used four-based spike metric similarity measures, Victor Purpura spike train metric, Van Rossum, Schreiber et al. and Hunter-Milton similarity measures [5,6]. In this work, we were able to investigate the direct effect of GABAA receptor on STN spiking activity, and our analysis provides also simple guidelines useful to search parameters that maximize irregularity.
